# Mitigating the impact of study-start delays in clinical trials for rare disorders: insights and lessons from a PKAN trial

**DOI:** 10.1186/s13023-025-04035-1

**Published:** 2025-11-11

**Authors:** Marleen M. G. Bracke, Sjoukje S. Polet, Mirjam Plantinga, Tom J. de Koning

**Affiliations:** 1 Expertise Centre for Movement Disorders, Department of Neurology, University Medical Centre Groningen, University of Groningen, PO Box 30.001, Groningen, 9700 RB The Netherlands; 2 Department of Genetics and Data Science Center in Health, University Medical Centre Groningen, University of Groningen, PO Box 30.001, Groningen, 9700 RB The Netherlands; 3 Department of Clinical Sciences, Section for Paediatrics, Lund University, Lund, Sweden; 4 Department of Neurology, Onze Lieve Vrouwe Gasthuis (OLVG), Amsterdam, The Netherlands

**Keywords:** Rare disorders, Progressive disorders, Clinical trials, Study initiation delays, Pantothenate kinase-associated neurodegeneration, PKAN, Qualitative interview study, Patient organisations

## Abstract

**Background:**

Rare disease clinical trials are notorious for complexities that frequently result in study-start delays. However, there is limited knowledge about how participants and researchers perceive these delays and what factors shape their experiences. A clinical trial in the rare, progressive disorder Pantothenate Kinase-Associated Neurodegeneration—the PKAN trial—encountered an unexpected delay. As this delay caused noticeable challenges for both PKAN participants and researchers, we sought to unravel their experiences in order to mitigate the impact of a study-start delay in future trials.

**Results:**

Fourteen semi-structured qualitative interviews were performed with PKAN participants (*n* = 9), represented by their caregivers or patient organisations, and researchers (*n* = 5) involved in the PKAN trial. During the delay, worries arose among some participants, which were directed towards the researchers. These participants expressed desperation to be included in the study in the early stages of the disease and held overly optimistic expectations for the PKAN trial. However, most participants did not experience a significant impact of the delay on their lives. On their side, the researchers mentioned the challenge of managing expectations while preserving hope. Most participants were satisfied with the communication; however, some participants highlighted concerns regarding the lack of transparency. Different interests between participants and researchers came to light during the delay. Some researchers advised providing background information on clinical trials, whereas all participants indicated that they did not need this information.

**Conclusions:**

Our study indicates that the delay in the start of the clinical trial had a significant effect on all the researchers and on some PKAN participants, especially those who urgently wanted the research to start, due to the pressure of the severe and progressive nature of the disorder. For these participants, there was a sense of discontent with how the researchers communicated, which made them feel that the researchers had different interests. This study also revealed that researchers had different perceptions of what information was needed than what participants wanted. To lessen the impact of such delays on participants and researchers, we recommend both honest and transparent communication and adjusting communication to meet participants’ needs. Close collaboration between participants, patient organisations and researchers can help achieve this goal.

## Background

Pantothenate kinase-associated neurodegeneration (PKAN), formerly known as Hallervorden-Spatz syndrome, is a rare neurodegenerative disorder with an estimated prevalence of 1–2 cases per 1 million individuals [1]. PKAN is an autosomal recessive disorder characterised by mutations in the PANK2 gene that lead to a disruption in pantothenate (vitamin B5) metabolism [2]. PKAN is the most common subtype of neurodegeneration with brain iron accumulation (NBIA) and is characterised by MRI changes in the basal ganglia [3]. Patients with PKAN exhibit progressive movement disorders, particularly dystonia, along with visual symptoms and cognitive dysfunction. The classification of PKAN distinguishes between two subtypes: classic and atypical. Classic PKAN typically begins with gait abnormalities in early childhood (mean age 3.4 years), and loss of ambulation occurs within 10–15 years of diagnosis. Atypical PKAN manifests in adolescents (mean age of 14 years) and often presents with speech difficulties. Atypical PKAN progresses more slowly and presents with neuropsychiatric symptoms such as behavioural difficulties and vocal and motor tics [4]. There is currently no FDA-approved disease-modifying treatment available for the fatal disorder PKAN [5].

Although each disorder affects only a small number of patients, rare disorders collectively affect approximately 400 million patients globally. Approximately half of rare disorders affect children, and most have a genetic aetiology and a progressive nature. At present, 95% of rare disorders lack an FDA-approved therapy [6]. However, fundamental research and clinical trials in rare disorders are being conducted to find new effective treatments. Between 1999 and 2017, clinical trials were conducted in 1535 rare disorders in the USA, E.U., and Japan. These trials investigated 1,539 different drugs in a total of 26,085 individuals [7]. Thus, rare disorder clinical trials involve a significant portion of the population.

Clinical trials for rare disorders are well known for their complexities, including the necessity for innovative trial designs and challenges in recruiting adequate eligible participants spread across diverse geographical locations. Moreover, the limited access to the vital resources needed to conduct a clinical trial, including funding, exacerbates this problem. This is often due to the small market sizes of orphan drugs, which lead to minimal economic impact and provide little return on investment [8]. Clinical trials that focus on implementing new therapeutic interventions frequently face obstacles related to regulatory approvals or recruitment difficulties [9–11]. Moreover, drug development faces lengthy timelines, as illustrated by central nervous system drugs, which take an average of 10 years to progress from submission to approval [12]. A previous evaluation revealed that more than half of the clinical trials in translational research face unforeseen average delays of 20 months, in addition to an estimated average trial duration of 35 months [13]. Therefore, obstacles in clinical trials for rare disorders result in delays in the start of clinical trials [14], yet the impact of the delay on study participants and clinical trial teams remains unknown.

Preclinical evidence shows that a vitamin supplement called 4’-phosphopanthetheine (4’-PPT), a downstream metabolic product of the enzyme lacking PKAN, can rescue the disease phenotype in animal and human cell PKAN models [15, 16]. In 2019, the first clinical trial with the study product 4’-PPT started in the USA (Clinicaltrial.gov: NCT04182763). Both clinical trials were part of a collaborative effort, and the development of 4'-PPT resulted from this partnership. Different types of clinical trials were conducted at both locations, testing various administration methods and dosages of 4'-PPT. In September 2021, a Dutch phase II clinical trial started with 10 Dutch and Belgian PKAN patients (hereafter referred to as the PKAN trial). This research focused on the absorption of different dosages of the study product by measuring several parameters in the participants’ blood while monitoring safety and tolerability. An academic grant (ZonMw) enabled the researchers to initiate this clinical trial, in line with the goal of affordable orphan drug development in a nonprofit setting [17]. In rare disorders such as PKAN, with predominant central nervous system involvement and heterogeneity in clinical manifestations and natural history, substantial challenges in study design and methodology exist during clinical trial development [18]. The Dutch PKAN trial also faced many challenges and could only start after several unforeseen delays. The delay of the PKAN trial caused several aversive reactions and intense emotions in participants, their caregivers, and patient organisations, as well as in researchers. Since delays frequently occur in clinical trials targeting rare disorders but, limited knowledge is available about how participants and researchers perceive these delays and what factors shape their experiences, this qualitative study was initiated to unravel the experiences of participants and researchers in the PKAN trial and mitigate the impact of a study-start delay in future clinical trials.

**Fig. 1:**
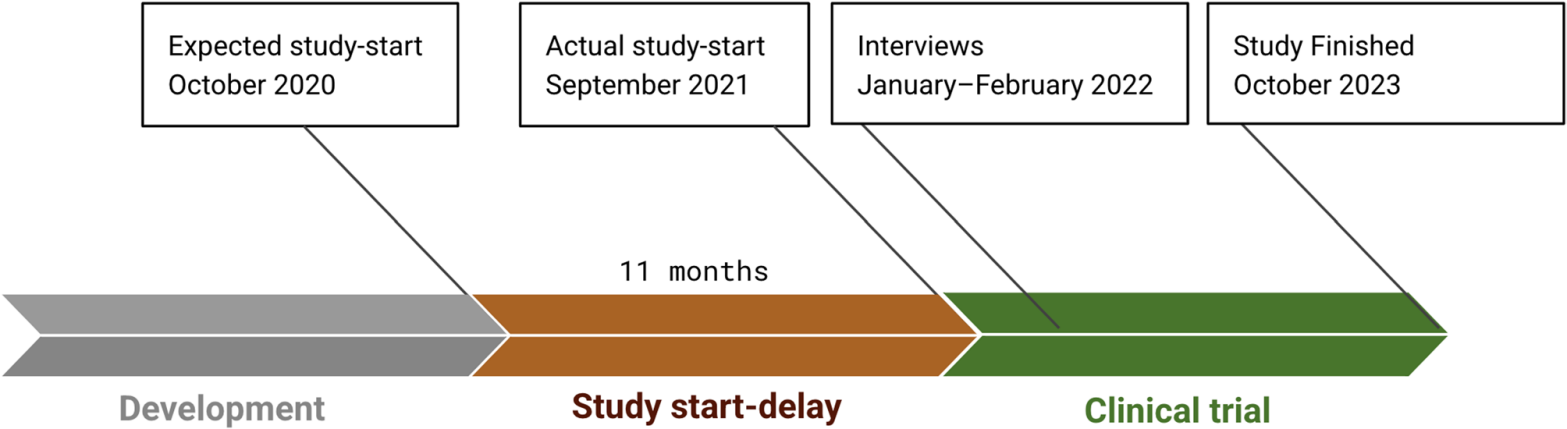
Overview of the study timeline

Figure 1 presents an overview of the PKAN trial timeline. Some future participants had heard about the preclinical results several years before the start of the study during a presentation on a patient day. At that time, there were discussions about a possible future clinical trial in humans, although there was no certainty that the trial would proceed, nor were any dates provided. Owing to the nonprofit nature of the research, securing the necessary funding presented challenges during this stage, referred to as the “developmental phase” (illustrated in grey in Fig. 1). Through a patient organisation, some future participants were involved in fundraising efforts for the trial.

After the developmental phase was completed and the protocol was finalised, the clinical trial was reviewed by the Dutch National Ethics Committee (CCMO) because of the inclusion of children in the PKAN trial. In accordance with Dutch regulations, the food supplements and vitamins used in such interventions are classified as pharmaceutical compounds and must adhere to the same regulatory standards as the medications used in clinical trials. The CCMO provisionally approved the protocol based on the information available at the time. The committee requested additional details regarding the stability of the vitamin product. The documents submitted by the clinical trial team were deemed insufficiently detailed, necessitating further stability testing before full approval could be granted. In combination with supply chain disruptions, these additional requirements led to delays. The occurrence of such delays highlights the challenges faced by even experienced researchers in investigator-initiated trials. This period between the published expected start date and the actual start date of the PKAN trial, referred to as the “study start delay” (illustrated in orange in Fig. 1), resulted in an 11-month delay.

Since the provisional approval, including an expected starting date, was already published on the website of CCMO and interpreted by some as a full approval, this delay was unexpected for the participants who had read this announcement and led to frustration. Some participants had inquired about the progress of the clinical trial to their treating clinician, which also resulted in a lack of clarity about the exact start date of the study. Others who had previously participated in the developmental phase through fundraising were also not aware of the delay in the expected start date. Finally, some participants were in contact with participants from the US, where the trial had already begun, and closely monitored developments in this clinical trial. This also raised many questions about why the Dutch PKAN trial could not start. The researchers attempted to communicate with the participants and their caregivers about the delay of the PKAN trial. Most communication about the progress of the PKAN trial, including unexpected delays, took place via newsletters. The newsletters were composed by the researchers, reviewed by the CCMO, and disseminated to participants through the patient organisations via email and social media. In the Netherlands, two patient organisations provide support to PKAN patients. One is solely dedicated to PKAN patients, while the other serves all patients diagnosed with NBIA, of which PKAN is a subset. Both patient organisations were involved in the PKAN trial.

It is crucial to recognise the fundamental differences in the values and motivations of the different stakeholders in rare disorder clinical trials, which often come to light in challenging situations such as a study start delay or a sudden trial termination [19]. An early-phase clinical trial, such as the PKAN trial, is designed to investigate safety and dosage, which differs significantly from the purpose of clinical care, which is to provide therapy in the patient’s best interest. Participants, or those providing consent for them, autonomously decide whether to participate in clinical trials. However, this informed consent may be distorted by various misconceptions [20]. A commonly cited motivation for patients with a rare disorder for which no treatment is available to participate in a clinical trial is the potential for personal benefit [19, 21]. In this context, a *therapeutic misconception* exists when individuals believe that research aims to benefit the individual rather than produce generalisable knowledge [22]. Similarly, clinicians may have similar misconceptions about the purpose of the clinical trial [23]. In addition, when participants overestimate benefits or underestimate risks, there is *therapeutic misestimation* [24]. Both misconceptions and misestimations should be recognised and handled to protect trial participants in their decision to enrol in a clinical trial [22, 24]. However, distinguishing these misconceptions from *hopes for the best personal outcome*—a phenomenon called *therapeutic optimism*—which should always be tolerated, is essential. Hope helps individuals to cope with existential threats, including their ability to cope with their progressive severe disorder [25]. Researchers are, in essence, driven by the desire to advance knowledge and apply this knowledge to the development of medical treatments. Moreover, clinicians must exercise their judgement and skills in the patient’s best interest. Representatives from patient organisations essentially value the voice of patients [26]. To resolve conflicts, recruit trial participants, build trust, and accelerate new therapies, numerous studies have stressed the importance of actively engaging patients and patient organisations in rare disorder research aimed at potential new therapeutic interventions, from an early stage onwards [8, 9, 14, 26, 27]. 

The objective of this study was to explore how patients, representatives, and investigators perceived delays in the start of the PKAN trial, and what lessons can be drawn for future rare disease trials.

## Methods

### Design

In-depth, semi-structured interviews were conducted with the participants of this qualitative study. The topics explored during the interviews were based on the research experience of the research team involved and supplemented by an extensive literature search. In this study, we collaborated with a representative from the NBIA patient organisation who was not directly involved in the disagreements arising from the delay. Within the research, the NBIA representative provided insights from the perspective of PKAN participants, and we held multiple meetings to discuss the clinical relevance of the interview guide and evaluate the topics. In addition, PKAN participants were offered the opportunity to contact the representative if they had any concerns or wished to discuss their participation in this qualitative study. Furthermore, to guarantee clinical relevance and methodological robustness, several researchers who were not involved in the PKAN trial provided input on the interview guide. During the interviews, we discussed topics related to general aspects of clinical trials for rare disorders, including *motivations*,* clinical trial participation*,* hopes and expectations*,* patient organisations and peer support*,* information and communication*, and *relationships*. These were followed by topics related to the study-start delay, including *experiences*,* coping*,* communication and information*, and *suggestions for improvements*. The study protocol was approved by the Medical Ethics Review Board of the University Medical Centre Groningen (RR number 202200110). We obtained informed consent from all participants in this study.

### Data collection

We invited all stakeholders involved in the delay of the PKAN trial. Table 1 provides an overview of the participant categories in the study: patients (or their representatives) and researchers. Invitations were sent by email. Non-responders received one reminder, and, upon request, considerations were further discussed by telephone. In the patient participants group, the invitations were addressed to the caregivers, who included parents, personal care attendants, or other family members, and to representatives from the patient organisations, since they were primarily involved in the PKAN trial. Among the twelve individuals approached in this category, nine participated. We invited PKAN participants to accompany their caregivers, but their involvement was often hindered by factors such as young age, cognitive impairments, and dysarthria. Despite these limitations, three PKAN participants participated in the interviews. In one interview, one caregiver represented two sibling PKAN participants. One representative from the NBIA patient organisation, who was not involved in the collaboration for this study, participated. The researcher participants included all the researchers involved in the PKAN trial, who were not involved in the current qualitative study, including laboratory and clinical researchers, complemented by the experiences of a representative with ample experience in rare disorder clinical trials from the study funder ZonMw. All five individuals approached within this group participated in the interviews.

Owing to the rarity of the disease and the limited number of researchers involved in this nonprofit study, the number of potential participants was obviously small. Nevertheless, the group reflected the Dutch PKAN cohort in terms of age, disease stage, and involvement with the trial, which makes it reasonably representative. All researchers involved in the PKAN trial also participated in this study, ensuring that the perspectives of the entire research team were captured. We therefore judged the sample to be the best possible given the circumstances to address the study aim, while of course acknowledging that the limited number of participants restricts the generalisability of our findings.

All participants provided signed informed consent to participate in the qualitative study. All the interviews were conducted by one interviewer (MB/the first author), who later became involved in the PKAN trial as a researcher responsible for data collection, but was not involved during the delay. All the interviews were conducted online (via Microsoft Teams) in January and February 2022. Each interview lasted between 32 and 112 min (with a mean duration of 64 min).

**Table 1:** Composition of interviewed study participants

Category	Patient participants (*n* = 9)	Researcher participants (*n* = 5)
Individual caregiver (representing one absent PKAN participant)	*n* = 3*	–
Two caregivers (representing one absent PKAN participant)	*n* = 1*	–
Individual caregiver (representing two absent PKAN participants)	*n* = 1*	–
Individual caregiver accompanying one PKAN participant	*n* = 3*	–
Representative from a patient organisation	*n* = 1	–
Clinical researchers involved in the PKAN trial	–	*n* = 2
Laboratory researcher involved in the PKAN trial	–	*n* = 1
Researcher involved in the developmental phase	–	*n* = 1
Representative from the funding organisation	–	*n* = 1

n = number of participants; * represents the number of PKAN participants present or represented

### Data processing and analysis

The interviews were audio and video recorded, with participants’ consent, and transcribed verbatim for further analysis. We then anonymised the transcriptions and deleted the recordings. To ensure coding consistency and high inter-coding reliability, the transcripts were analysed thematically by two independent researchers (MB and SP) using the qualitative analysis software package ATLAS.ti. The second researcher (SP) was not involved in the PKAN trial. We conducted multiple meetings to identify themes and achieved consensus in coding by discussing discrepancies. All participants approved an abbreviated summary of their interview, categorised into topics.

## Results

Six key themes emerged from the interviews: (1) Hopes, Expectations and Motivations, (2) Impact of the Delay, (3) Coping with the Delay, (4) Communication regarding the Delay, (5) Collaborations and Differing Interests, and (6) Recommendations for Future Research. Below, we provide an in-depth discussion of each theme. For all the themes, the results from patient participants and researcher participants are presented separately. To avoid confusion, we refer to patient participants as ‘participants’ and researcher participants as ‘researchers’ throughout the text.

**Table 2:** Quotations by theme

Theme	Quotation
Hopes, Expectations and Motivations	1. P5: “There’s nothing; the only thing you can cling to is hope.” 2. P6: “That’s the only straw we have to grasp at. Otherwise, our child will die.” 3. R12: “It’s just very promising in animal models. I think if you repeat that a lot, people will think: If it’s so promising in a mouse, then it has to be promising in a human too.”
Impact of the Delay	4. P6: “Every minute was a minute for us, yeah? That shouldn’t be forgotten. Time is our greatest enemy. I can’t say that often enough. So any delay is just horrible.” 5. R11: “It has had a huge personal impact on me. I’ve been really upset. Sometimes so much so that I thought: I don’t know if I can continue with this.”
Coping with the Delay	6. P2: ‘’You’re going to start with a drug that you administer to the body. So there must be a reason for the delay. And then you just wait. It’s not just some package from bol.com that you’re waiting for.‘’ 7. P15: “How the hell can you spend 14 months trying to figure it out? In my view, that says it’s just not a priority. And I personally blame them for that.” 8. P14: “I think contact with fellow sufferers is fundamentally more important. Because you live in the now. As long as there is no medicine, you need each other, to not be alone.” 9. R11: “And it led to tensions within the team. Because you really have to be very much on the same page. When you’re in trouble like that (…) What do you tell? What don’t you tell? And what’s really going on? And it’s a very complex subject.”
Communication Regarding the Delay	10. P6: “The lack of information, that’s going to make you come up with ideas like that. And it was never completely transparent, towards the patient associations and towards parents, and that kind of eats at you. What is the reason for that?” 11. R10: “No, not in terms of timing. That was more on us. Since we did indicate, we expected to be able to start sooner rather than later. It was a bit tight.”
Collaborations and Differing Interests	12. P6: “Swallow three times, push away your tears and move on. Because there’s nothing we can do. A bit of anger too, dammit, frustration and anger come together. But always still reminding yourself: *people work hard*,* mistakes have been made*,* but people work hard*. And we just have a different stake here. And occasionally still getting the feeling that they don’t see the importance for our child.” 13. R12: “Ultimately, the same interest, of course, is being able to treat PKAN patients, but we also think about the long term. Can we keep the drug available in the long run, if it works? Or maybe much longer trials are needed. Or maybe in a different setup, or something else. But patients just want their child to get better as soon as possible. Yes, I think those interests are partly similar, but partly very different.”
Advice for Future Research	14. P3: ‘’Honestly saying: ‘we are delayed’. That would have been the right thing to do. Ok, if that’s the case, then so be it. We were informed, they let us know they were going to start. They didn’t forget us, that was the main thing.” 15. R10: “You also want to involve the people, you want to recruit of course, that went very fast now, and that has never happened so quickly in any study. That’s thanks to the fact that we had them involved.”

### Theme 1: hopes, expectations and motivations

#### Patient participants

For most participants, the PKAN trial represented their first opportunity to participate in a research study, offering a sense of hope. All the participants hoped for positive clinical effects, such as improved movement, better quality of life, pain relief, and better speech and hand function. The trial symbolised their hope for a better future perspective by slowing down or stopping disease progression. Some even hoped for a cure, whereas others considered any insights into future treatments good news. While they recognised that their hope might be overly optimistic, they emphasised its importance as a source of endurance during challenging times (Quote 1, Table 2). A few participants mentioned that they expected the same outcome that they hoped for.

The sense of urgency and expectations differed greatly within the patient participant group. On the one hand, the majority of participants expressed low expectations for the upcoming trial. They wanted to remain realistic, and the realization was that improvement might not be possible, as brain damage is irreversible. Many of them demonstrated an understanding of the complexities of drug development, acknowledged the unknown efficacy of the drug, and noted the lack of available treatments since diagnosis. However, given the progressive nature of the disease, these participants still felt pressure to commence the research as quickly as possible.

In contrast, some participants emphasised that the research was their only lifeline, a matter of life and death (Quote 2, Table 2). They wanted to enrol their child in the PKAN trial as early as possible, when symptoms were still relatively mild, compared to end-stage disease. They stressed that time is a major enemy of this progressive, life-threatening disorder. This urgency made them desperate to start the PKAN trial as soon as possible. These participants addressed their overly optimistic expectations after the study’s announcement, where researchers had shared promising results from animal research. They highlighted a difference in the perception of time between researchers and participants. They also mentioned their tendency to interpret information according to their hopes, which they called “you hear what you hope to hear”. Furthermore, expectations were high at that time due to the initiation of US research and the reports from these participants that were shared on social media platforms such as Facebook and WhatsApp.

The participants consistently expressed their motivation for enrolling in the PKAN trial, with personal benefit being a common driving factor. Participating in the PKAN trial provided hope for a better future perspective. They felt that they had nothing to lose, considering the lack of effective treatment and the expected safety of the study product. While most participants did not actively consider the potential occurrence of side effects, they did express the freedom to discontinue their participation in the research at any time. Another significant motivation shared by many participants was their desire to contribute to developing a treatment for future patients. For some, this contribution was their primary motivation. Additionally, some participants saw the opportunity to meet other individuals with PKAN as a significant motivating factor for joining the clinical trial.

#### Researcher participants

While most researchers had no specific expectations, they did anticipate that the study product would be safe and well-tolerated. All the researchers discussed their hopes from two perspectives: as professionals and as humans. At the human level, they wished for improved quality of life and a slowdown in disease progression. From a professional standpoint, they hoped to see positive results in the outcome measures. All researchers were motivated by the chance to apply their expertise to benefit PKAN patients. In addition, they discussed being driven by the severity of the disease, their passion for the work, and the shared goal of developing an affordable treatment.

Many researchers have expressed concerns about the unrealistic expectations held by some participants. They attributed these unrealistic expectations to factors such as a deep desire for a better future for their child, differences in interpretations of scientific language (Quote 3, Table 2), lack of knowledge about drug development, conversations with participants in the USA, and the presence of a competent researcher who instils hope in participants. Multiple researchers have discussed their efforts to manage expectations by emphasising that the clinical effect is unknown and was not the focus of this study. At the same time, they did not want to take away any hope.

### Theme 2: impact of the delay

#### Patient participants

The impact of the study-start delay varied among participants. For most participants, the delay had no significant impact. Some participants experienced disappointment rather than significant distress when receiving updates about the delay. Some participants expressed feelings of occasional helplessness, whereas others stated that they perceived minimal impact from the delay, recognising their inability to influence it. Some participants were unaware of trial preparation’s process or the setbacks causing delays until just before the start of the PKAN trial.

However, some participants experienced a significant impact of the study-start delay. The opportunity to participate in this study felt like a matter of life and death to them, leading to feelings of desperation when delays occurred. They observed that their children declined during the waiting period for the study and felt the urgency to start as soon as possible when symptoms were less advanced (Quote 4, Table 2). They highlighted that the progression of time felt agonisingly slow during the waiting period, and they experienced a sense of powerlessness. They described the delay as a stressful period marked by emotions of sadness, anger, and frustration, during which they experienced physical and emotional consequences of stress.

#### Researcher participants

The delay significantly impacted all the researchers and was experienced as a rough and frustrating period. The cause of the delay was highly frustrating. Some participants mentioned experiencing a loss of control and that dealing with the delay consumed considerable time and energy. Most researchers felt deeply upset, and some felt intimidated by the reproachful emails they received. However, they expressed their understanding of the frustrations of those affected by the delay. Researchers mentioned their fear of the consequences of the delay. Some mentioned an influence on their private lives. Most researchers expressed a desire to discontinue their involvement in their research activities in the PKAN trial (Quote 5, Table 2).

### Theme 3: coping with the delay

#### Patient participants

The way participants coped with the delay differed among the participants. Most participants refrained from acting as they perceived that it would not change the circumstances and trusted that there would be a valid reason for the delay (Quote 6, Table 2). They waited patiently and hoped for the research to commence soon. They found the updates from the patient organisation sufficient, trusted the researchers that they were making progress, and did not want to bother the researchers with questions. Most participants sought information from USA participants about their experiences with the trial. The participants, unaware of the delay, reported that they would not have taken any action even if they had been aware of it.

Some participants, however, initiated conversations with the researchers, and some of these participants expressed frustration in grieving emails directed to the researchers. They primarily directed their frustrations toward the reasons for the delay and how the researchers caused delays due to mistakes they made (Quote 7, Table 2). They mentioned that the process could have been faster and more efficient. A few family members of participants became proactive in efforts to speed up the start of the study as board members in a patient organisation. Some participants attempted to participate in the US clinical trial.

By the time of the interviews, the PKAN trial had commenced. Most participants who had previously expressed overly optimistic expectations upon the announcement of the clinical trial now reported having no or uncertain expectations. They cited various factors that contributed to their lowered expectations, such as explanations from researchers emphasising that resolving PKAN is complex, self-protective measures following disappointments, and the disease progression during the months of delay. Since the start of the study, most participants have expressed positive sentiment regarding their relationship with the researchers. They appreciated the researchers being approachable, responding quickly, and being friendly, which made them feel comfortable and trusting. Several participants shared their experiences of forming close bonds with other participants since the PKAN trial, with some emphasising that this peer support held greater significance for them than the actual effect of the study product (Quote 8, Table 2). During the interview, a participant who was unable to participate in the PKAN trial because of symptom progression resulting from the delay, spoke of a shift in mindset—moving from frustration to acceptance and living day by day.

#### Researcher participants

The unpleasant interactions with participants triggered difficult discussions within the team, leading the researchers to anonymize their communications out of fear of potential consequences (Quote 9, Table 2). A barrier emerged between the parties involved, making effective communication and mutual understanding more challenging. All the researchers mentioned their agonised efforts to manage the setbacks. They worked overtime extensively during the period of the delay. Since the budget for the trial came from a research grant, there were no means to hire extra personnel. Additionally, in the absence of a preexisting clinical trial framework for PKAN, setting up the study required substantial effort, extensive investigation, and problem-solving. The researchers remained motivated to continue their work because they recognised the importance of studying this severe disorder to aid patients. Additionally, the majority highlighted the motivating and inspiring atmosphere within the team.

Once the PKAN trial was approved and started, the relationship with most participants normalised, and the collaboration with patient organisations proceeded smoothly. Patient organisations played a significant role in the fast recruitment of participants in the clinical trial.

### Theme 4: communication regarding the delay

#### Patient participants

The way participants experienced the communication and information provision varied significantly. On the one hand, most participants felt that they were kept well-informed about the upcoming trial throughout the process. They appreciated receiving updates to know that work was in progress. The cause of the delay was clear to them. They felt well-informed, which instilled confidence that the delay was being addressed. These participants spoke positively about the newsletters, expressing their satisfaction with the regular updates on progress and the clarity of information. At the time of the delay, some participants were not involved with any patient organisation, so they did not receive any newsletters.

On the other hand, some participants highlighted concerns regarding the lack of information and transparency, which led to uncertainty about progress and the exact reasons behind the delay (Quote 10, Table 2). Some participants began to question what interests were at stake for the researchers. Furthermore, they reported receiving conflicting information from various sources, such as newsletters and interactions with US and Dutch researchers. This influx of inconsistent information further added to their frustration. These participants mentioned the good intentions conveyed with the newsletters, but they felt that they did not offer much substantial information and noted that the newsletter was unfortunately timed, as it was released just before Christmas.

The way the delay was experienced frequently influenced how the participants perceived the communication. Those who felt a significant impact from the delay expressed a heightened need for information and held higher expectations regarding communication from the researchers. Simultaneously, most participants highlighted that their experience with communication regarding the delay was a central factor in how they perceived the delay itself.

The researchers organised an information meeting once the PKAN trial was definitively set to commence, based on a recommendation from a patient organisation. The delay was discussed, and practical information regarding the PKAN trial was provided. Almost all the participants attended and were enthusiastic about this online meeting. Some participants only heard about the delay for the first time that evening. They were pleased that the study was about to commence, and they significantly appreciated seeing faces and engaging in conversations. Additionally, some participants expressed appreciation for the chance to ask questions of the patient organisation afterwards.

#### Researcher participants

Most researchers believed that their overall communication was accurate and well thought-out, despite encountering numerous challenges. They tried to provide nuanced information, fearing that information might be interpreted differently otherwise. They reflected on their initial communication as overly optimistic about the progress of preparations, including the expected start date (Quote 11, Table 2). They also found it challenging to explain all aspects of the delay due to its complexity and confidentiality requirements. As a result, the information in the newsletters lacked transparency. From their perspective, the causes of the delay seemed more straightforward to outsiders, and explaining every detail was overly time-consuming. Adding to these complications was the problem of controlling public information, particularly on social media, which could spread confusing or mixed messages. The lack of clarity about who should communicate specific information amplified this problem, leading to additional confusion and uncertainty. When disseminating a newsletter, several researchers were apprehensive about potential adverse feedback. The researchers reflected on the primary mode of communication, newsletters, as not ideal due to the absence of nonverbal cues. Moreover, following unpleasant interactions, the researchers anonymized emails, making the communication more impersonal. The timing of the newsletter distribution was another concern, with many researchers identifying the decision to send it just before Christmas as a significant misstep.

Some researchers highlighted the pressures tied to organising the information evening, expressing concerns that any misstep could be used against them. However, the evening was positively received, in part because it provided a platform to share the uplifting news that the study would begin and because all participants were present, including those participants who entered the discussion without prior knowledge of the setbacks.

### Theme 5 collaborations and differing interests

#### Patient participants

Within the patient participant group, there was a variation in the level of trust towards the researchers, which mainly was correlated with how the delay was experienced. Most participants had confidence in the competence of the researchers and trusted that they were doing everything possible to initiate the research as quickly as possible. In addition, they expressed confidence that there would be a sound reason for the delay. However, some participants expressed concerns about potential conflicts of interest due to the researchers’ lack of honest and transparent communication. In their opinion, participants felt a greater sense of urgency than researchers did (Quote 12, Table 2). They brought the researchers’ competence into question. They indicated that the process could have been faster and more efficient and that the researchers made mistakes. Although some participants experienced these concerns and frustrations, all indicated that they had no personal issues with any researchers.

Throughout the delay, most participants experienced considerable support from patient organisations, which substantially mitigated their burden by undertaking proactive initiatives. Most relied on these organisations to pressure the researchers to accelerate the start of the research and keep them informed. During this period, it was challenging for the patient organisations to meet the needs of their patients. The researchers did not always provide new information in the newsletters, leading the patient organisations to decide against distributing it.

#### Researcher participants

During the delay, researchers had mixed feelings about the involvement of patient organisations, seeing their role as both stimulating and challenging. They were delighted with the assistance from patient organisations in disseminating information to participants and providing constructive advice. However, some researchers emphasised that they lacked the training to involve patient organisations effectively. The researchers struggled with communication and failed to establish clear guidelines regarding the roles of the patient organisations. Researchers mentioned the complexity of collaboration when members of a patient organisation were emotionally involved due to having family members with the disorder. While this emotional involvement fuelled their motivation, it posed a challenge to maintaining an objective perspective. The researchers felt that they had to justify their actions and negotiate terms. In this collaboration, the researchers pointed out a conflict of interest: parents want immediate treatment for their child, whereas researchers are concerned with long-term outcomes. They emphasised that the final aim remains the same – the treatment of PKAN patients (Quote 13, Table 2).

### Theme 6 recommendations for future research

#### Patient participants

Participants provided various recommendations on communication during delays, highlighting the importance of transparency and honesty when communicating about delays and urging against overly optimistic portrayals. They requested clear, accessible explanations. Additionally, participants indicated that mistakes should be acknowledged (Quote 14, Table 2). With respect to content, participants indicated a desire to receive updates on several specific topics: the anticipated start date (acknowledging potential changes), reasons for delays, interim results, findings from related studies, and information about future research opportunities. They did not indicate a desire for background information on scientific research. Additionally, they showed no interest in receive further information about the purpose of examinations during the home visits, including video recordings, despite most participants being unaware of the intended use of the video, particularly since it was not used to evaluate clinical efficacy. All the participants indicated that their primary concern centred on the initiation of the specific study, which represents a potential opportunity for their child’s future. Some participants wanted a dedicated point of contact to receive information about future studies. Most participants would like to receive information in newsletters via email at agreed-upon intervals from an early stage. However, they prefer online meetings or phone calls as more suitable alternatives when adverse reactions or numerous questions arise. Physical meetings are seen as burdensome additions to their already busy lives.

The participants emphasised the importance of collaborating with a patient organisation to co-create content and disseminate information, thereby ensuring that they receive relevant information and newsletters at suitable times. In this collaboration, patient organisations can represent the participants and are more approachable for participant feedback. Some participants suggested that physicians should inform new patients about the existence of patient organisations.

#### Researcher participants

All researchers provided recommendations on communication in future investigations, emphasising two main objectives: (1) preventing potential disagreements and (2) encouraging patients to participate in the research, thereby accelerating the recruitment. Researchers agree that it is only possible to achieve these goals if participants trust the researchers, a relationship that researchers can build by strongly emphasising transparency, honesty, and open communication.

Within this context, the researchers advise that expectation management is an essential topic. They advise setting realistic expectations about the status of preparations, the uncertainties around clinical effects, and the potential burdens the study may entail. Furthermore, researchers recommend carefully considering how participants interpret information and encourage the repetition of key messages. Their advice suggests caution when specifying an expected start date. They recommended communicating that work is in progress and that the research commences ‘eventually’, rather than specifying a particular date. Additionally, some suggest providing background information on what it means for patients to participate in a clinical trial. Topics that should be addressed include an explanation of what a clinical trial entails, a basic understanding of drug development, and the implications of participating in research for both an individual and a group.

With respect to communication with participants, researchers advise a leading role for clinical researchers. In addition, they recommend allocating funding to provide personalised attention during a delay. Most researchers recommend working together with patient organisations to disseminate information as needed. They highlight the importance of keeping all stakeholders informed at agreed-upon intervals. Many researchers suggest using newsletters for communication and organising regular face-to-face or online meetings from an early stage.

Many researchers have highlighted the value of teamwork between patient organisations and researchers. By engaging participants, one can accelerate recruitment (Quote 15, Table 2). Most researchers consider the value of clear expectations for patient organisations by promising realistic goals and agreements on suitable roles. Researchers and patient organisations should schedule regular meetings. In addition, an objective person should accompany the patient organisations in essential conversations with researchers. Researchers and patient organisations should consider undertaking relevant training for a successful collaboration.

## Discussion

The complexities of rare disorder clinical trials are notorious, which often result in study-start delays. Overcoming the challenges associated with designing clinical trials for rare disorders can sometimes be achieved through effective partnerships with rare disease experts, seeking regulatory and biostatistical guidance, and early engagement of participants and their families in the research process. Furthermore, a paradigm shift in the regulatory process is needed [18]. However, if delays do occur, we must learn how to best manage them to mitigate their impact. This qualitative study describes experiences, factors that contributed to these experiences, and lessons from participants and researchers in a delayed start of a clinical trial for PKAN. The delay represented a challenging period for all researchers and some participants. Several aversive reactions and intense emotions arose in participants who were directed towards the researchers. However, this study indicates that most participants did not experience a significant impact on their lives due to the delay. Despite the challenges during the delay, relations were normalised once the trial commenced. Our findings identify three key factors that contribute to differing experiences during the delay, along with strategies to mitigate the impact of a delay. The three factors are as follows: (1) Hopes, expectations, and motivations, (2) Communication regarding the delay, and (3) Collaborations and differing interests.

The expectations and sense of urgency for the commencement of the PKAN trial varied among the patient participant group. Most participants expressed low expectations for the upcoming trial. These participants felt pressure from the progressive nature of the disease to commence the research as quickly as possible. However, this pressure did not lead them to experience a significant impact from the study delay. In contrast, some participants held overly optimistic expectations for the PKAN trial after hearing encouraging news from the researchers and interpreting information based on their hopes. For these participants, the potential of this research seemed to be their only lifeline. These participants expressed desperation to be included in the PKAN trial while their symptoms were less advanced. Consequently, these heightened expectations and intense sense of urgency led them to feel a profound impact from the delay. In studies reporting clinical trials in paediatric disorders, parents experience an increasing urge to participate when their child has more severe symptoms [19, 21]. Owing to the rapid progression and debilitating symptoms of PKAN, a sense of urgency is felt from an early stage of the disease. All the participants hoped that participating in the PKAN trial would provide a better future. This hope became their anchor, providing them with the strength to navigate the arduous journey ahead. Researchers expressed concerns regarding the unrealistic expectations held by some participants. They attempted to manage these expectations while preserving hope. Hope should always be tolerated [24], even encouraged [26], since hope empowers individuals to cope with existential threats, including the ability of patients to cope with their progressive severe disorder [25]. Most participants reported their primary motivation for enrolling to be a personal benefit. The focus on personal benefit seemed to reflect emotional interest rather than a misconception of the trial goals. This finding was similar to previous findings in rare disease clinical trials [19, 21, 28].

The impact of both the contents and methods of communication became evident during the study-start delay, when both participants and researchers reported unpleasant interactions. The way in which participants experienced communication and information provision varied significantly. Most participants were satisfied with the information they received from the researchers through newsletters. However, other participants reported that the communication from the researchers was not transparent, and they received inconsistent information from different parties, leading to ambiguities. This study revealed that the informational needs and expectations regarding communication from researchers were greater when the participants experienced a significant impact of the delay. Simultaneously, most participants highlighted that their experience with communication regarding the delay was a central factor in how they perceived the delay itself. Researchers cited several communication challenges they encountered during the delay, such as differences in interpretations of scientific language.

There was consensus between both participants and researchers on multiple key recommendations for future research. First, to prevent potential disagreements and motivate patients to participate in a study, researchers must communicate about any delays with honesty and transparency. Moreover, they should disseminate realistic information, particularly regarding anticipated start dates, knowing that unexpected delays do occur in most clinical trials for rare disorders. Finally, patient organisations should be involved in creating and disseminating information. Similar to what we observed in this PKAN trial, the participants in a Duchenne study that was unexpectedly stopped before the closing date wished for more communication about decisions and the path forward [19].

In contrast, some recommendations revealed discrepancies between the researchers’ suggestions and participants’ desires. After unpleasant interactions, researchers anonymized their emails, making the communication impersonal. For future studies, the researchers recommended dedicating time and effort to building relationships with participants by engaging with one another at an early phase, ideally through in-person meetings. However, owing to their busy personal lives, participants prefer to be informed via email. Similar to the researchers’ suggestions, in a study with Duchenne patients, Paey et al. (2014) reported that establishing “family-like” relationships between participants and researchers notably improved participants’ overall well-being. This finding proved particularly crucial when participants experienced feelings of powerlessness during the abrupt termination of that trial [19]. Moreover, some researchers have recommended providing background information on what it means for patients to participate in a clinical trial. However, the participants did not express a desire to receive this information, as their primary concern focused on the commencement of the specific study, which represented a potential opportunity for their child’s future. In line with the researchers’ recommendation, other studies on clinical trials involving rare diseases have suggested providing participants with information to support informed decision-making, for example, about the trial process and potential barriers to long-term access to the drug [21, 29]. Given the discrepancies we found between the researchers’ perceptions of relevant information and the participants’ views on what information they wanted to receive, the participants’ desires for information provision, and the impact of this information mismatch should be investigated further.

Different interests between participants and researchers came to light during the delay. Participants wished for immediate treatment, whereas researchers were more concerned with safety and long-term outcomes. Due to a perceived lack of clear communication, these participants felt that researchers did not recognise the urgency. Similar conflicts of interest have been reported in other clinical trials targeting rare diseases [19, 26, 27]. However, the goal shared by all parties in a research setting is the development of the future treatment and well-being of the participants. To achieve teamwork between researchers and participants, it is necessary to promise realistic goals and make agreements on suitable roles. Collaboration between participants and researchers helps researchers better understand participants’ needs and interests, while participants can better understand the research process and logistics [20, 26]. Consistent with prior research, the PKAN researchers emphasised the need to train researchers to address the challenges inherent in conducting clinical trials for rare diseases [8, 27, 29, 30].

Given the limited understanding of the impact of delays in clinical trials, this study offers critical insights for researchers, clinicians, patient organisations and participants who are likely to encounter such challenges in the future. Through a qualitative research approach, we analysed and compared the experiences of both participants and researchers. This method facilitated a more profound comprehension of their distinct experiences, along with the factors that influence them, providing invaluable lessons to mitigate potential disagreements in upcoming studies. All the participants expressed satisfaction with the interview process, despite the sensitive nature of the topic. Furthermore, many recognised the significance of these interviews for guiding future research and enhancing communication within the PKAN trial.

This study has four main limitations. First, it is essential to acknowledge that the interviews took place when the clinical trial had already been underway for several months. This temporal aspect could impact the results obtained from the interviews. However, as the study start of the PKAN trial was time-consuming and already required significant time investments from both the participants and researchers, it would have been too burdensome to conduct the interviews during this already hectic period. Second, the interviewer (MB) tried to separate her role as an interviewer in this qualitative study from her role as a researcher in the PKAN trial, with a clarification given at the start of the interviews. At the end of the interviews, many participants seemed to rely on a preexisting relationship, commonly expressed as ‘good, I already know you’. A representative from the NBIA patient organisation was also involved in the study to bring in the participants’ point of view. Unfortunately, because of the possible confusion of roles and potential conflicts of interest, one patient organisation and the caregivers of one PKAN participant chose not to participate in this qualitative research, resulting in the loss of potentially valuable data. Third, although both groups were considered representative of their respective stakeholder categories, the number of interviews was not sufficient to ensure data saturation [31]. In the researcher group, experiences were relatively homogeneous, so core themes became identifiable even with only five interviews. The patient group was more diverse and included only one patient organisation, meaning that nine interviews were insufficient to claim full saturation. Nevertheless, both investigators (MB and SP) observed recurring key themes across participants in both groups. The limited number of interviews restricts the generalisability of our findings beyond the current cohort, although the sample was adequate to address the specific study aim. Finally, all the interviews were conducted and recorded via video calls (Microsoft Teams) due to logistical considerations and the COVID-19 pandemic. While this meant that connections were very occasionally lost, the recordings were convenient during the analysis phase as they made it possible to review the video and explore nonverbal cues further.

## Conclusions

A delay in the start of a clinical trial that offers hope to participants suffering from a severe, progressive disease is invariably challenging. Here, such a delay had a significant effect on all the researchers and on some participants, especially those who urgently wanted the research to start, owing to the pressure of the severe and progressive nature of the disorder. For these participants, there was a sense of discontent with how the researchers communicated, which made them feel that the researchers had different interests. This study also revealed that researchers had a different perception of what information was needed than what participants wanted. To lessen the impact of such delays on participants and researchers, we recommend both honest and transparent communication, and adjusting communication to meet participants’ needs, while recognising likely differences between participants in their informational needs and expectations regarding communication. Close collaboration between participants, patient organisations and researchers can help achieve this goal.

## Data Availability

To safeguard the privacy of our study participants, the research data are not publicly accessible. The interview guide (in Dutch) is available upon request from the authors.

## Abbreviations

PKAN: Pantothenate Kinase-Associated Neurodegeneration
NBIA: Neurodegeneration with Brain Iron Accumulation
FDA: Food and Drug Administration
USA: United States of America
EU: European Union
4’-PPT: 4’-phosphopantetheine
